# Vehicle-Assisted Techniques for Health Monitoring of Bridges

**DOI:** 10.3390/s20123460

**Published:** 2020-06-19

**Authors:** Hoofar Shokravi, Hooman Shokravi, Norhisham Bakhary, Mahshid Heidarrezaei, Seyed Saeid Rahimian Koloor, Michal Petrů

**Affiliations:** 1 School of Civil Engineering, Faculty of Engineering, Universiti Teknologi Malaysia, Skudai, Johor 81310, Malaysia; norhisham@utm.my; 2 Department of Civil Engineering, Islamic Azad University, Tabriz 5157944533, Iran; hooman.shokravi@gmail.com; 3 Institute of Noise and Vibration, Universiti Teknologi Malaysia, City Campus, Jalan Semarak, Kuala Lumpur 54100, Malaysia; 4 Department of Bioprocess Engineering, School of Chemical and Energy Engineering, Faculty of Engineering, Universiti Teknologi Malaysia, Skudai, Johor 81310, Malaysia; m.heydarrezaei@gmail.com; 5 Institute for Nanomaterials, Advanced Technologies and Innovation (CXI), Technical University of Liberec (TUL), Studentska 2, 461 17 Liberec, Czech Republic; s.s.r.koloor@gmail.com (S.S.R.K.); michal.petru@tul.cz (M.P.)

**Keywords:** structural health monitoring (SHM), drive-by damage detection, indirect structural health monitoring, weigh in motion (WIM), bridge weigh in motion (BWIM), vehicle bridge interaction (VBI)

## Abstract

Bridges are designed to withstand different types of loads, including dead, live, environmental, and occasional loads during their service period. Moving vehicles are the main source of the applied live load on bridges. The applied load to highway bridges depends on several traffic parameters such as weight of vehicles, axle load, configuration of axles, position of vehicles on the bridge, number of vehicles, direction, and vehicle’s speed. The estimation of traffic loadings on bridges are generally notional and, consequently, can be excessively conservative. Hence, accurate prediction of the in-service performance of a bridge structure is very desirable and great savings can be achieved through the accurate assessment of the applied traffic load in existing bridges. In this paper, a review is conducted on conventional vehicle-based health monitoring methods used for bridges. Vision-based, weigh in motion (WIM), bridge weigh in motion (BWIM), drive-by and vehicle bridge interaction (VBI)-based models are the methods that are generally used in the structural health monitoring (SHM) of bridges. The performance of vehicle-assisted methods is studied and suggestions for future work in this area are addressed, including alleviating the downsides of each approach to disentangle the complexities, and adopting intelligent and autonomous vehicle-assisted methods for health monitoring of bridges.

## 1. Introduction

Bridges are subjected to deterioration caused by detrimental factors, such as aging, fatigue, and corrosion that degrade structural capacity. Failure of civil structures may lead to catastrophic consequences in terms of human life and economic assets. Then it is of utmost importance to ensure the structural integrity of bridges to meet safety, durability, and serviceability requirements [1]. Structural health monitoring (SHM) is a highly active research area with promising technological implications to evaluate structural performance and to identify damage during the service life of a structure [2]. Health monitoring of structures is aimed to improve safety and reliability through (1) assessing the operational condition of a structure in a near real-time manner and providing an alarm for abnormal conditions, (2) evaluating the serviceability of structures immediately after occurrence of extreme loading conditions such as an earthquake or major vehicular collisions and (3) providing the owners with accurate information on a structure to decide about the most cost-effective repair and rehabilitation strategies in a certain civil engineering structure [3,4,5]. 

SHM is an interdisciplinary subject that incorporates knowledge and experiences from synergetic technologies to deal with the health assessment of structures. SHM approaches assume that damages affect the dynamic or static properties of a system [6,7]. SHM collects structural responses from several points through mounted sensors, analyzes data, and evaluates the health state of the structure. Strain and displacement of a structure are affected by damage under specific static loading. On the other hand, damage reduces stiffness and Young’s Modulus and consequently results in a change in modal parameters. Detectability of a damage detection system is highly influenced by the type and size of imperfection. 

Bridges are designed to resist load components such as dead, live, environmental, and occasional loads. Temperature, wind, and traffic are the most important operational load during the service period and they greatly affect the structural behavior of a bridge structure. The applied vehicular traffic load is a parameter that can be easily monitored and their effect is distinguishable from the induced environmental-based structural responses. There is a close correlation between the property of a bridge structure and the corresponding structural response. Hence, the establishment of a relationship between the applied traffic load and the extracted response signal is a valuable indicator to evaluate the structural health condition of a bridge structure [1]. The applied traffic load to a highway bridge structure depends on several parameters such as weight of vehicles, axle load, configuration of axles, position of vehicles on the bridge, number of vehicles, direction, and vehicle’s speed [8]. Though it is very desirable to evaluate the in-service performance of a bridge structure, it is difficult to measure the incorporated time-varying vehicular parameters [9]. 

Over the past decades many methods for identification of moving vehicles on bridges have been reported. O’Connor et al. [10] proposed a method to estimate the variation of dynamic load based on the static mass of a moving load on a bridge. Their method is named the interpretative method I (IMI); vehicles on a bridge are modeled analytically as a set of lumped mass linked with massless beam elements. Chan et al. [11] introduced another method to identify the moving dynamic load using a numerical model of bridges and the bridge-vehicle interaction. The proposed model was named interpretative method II (IMII) and was similar to the IMI technique. Most of the proposed inverse methods for identification of the applied force from the response exhibit significant fluctuation in the start and end of time history due to an ill-conditioned inverse problem. Law et al. [12] proposed using the regularization method to solve an ill-conditioned problem. 

A variety of techniques have been proposed for measuring moving vehicle loads on bridges [13]. Using instrumented vehicles is a traditional approach to measure live load on bridges [14]. Implementing this test is difficult, expensive and the extracted data are prone to bias since the experiment is limited to the instrumented vehicle. Henchi et al. [15] used analytical modeling of moving vehicles and bridge deck to calculate vehicular live load. However, using a dynamic model for extracting traffic load is subjected to error and imprecision. Weigh in motion (WIM) system developed for measuring vehicle weight data [16]. WIM is a system equipped with various sensors, digital cameras, and computers that is installed on a bridge structure. WIM measures the dynamic axle load of moving vehicles to obtain vehicle weight data. However, WIM is a fixed-location weight measurement device and measures the axle weight just when its wheels pass over the sensors. On the other hand, WIM system is expensive and not feasible for local roads. 

Deng et al. [17] proposed a direct method for the identification of axle load from bridge response. In their method modal parameters of bridges and the mechanical properties of vehicles are used to develop a vehicle–bridge couple system. The dynamic axle load can be determined from the influence surface and superposition concepts. In a companion paper, Deng and Cai [18] evaluated the numerical model of an existing bridge by passing two trucks across in parallel. The result obtained from the experimental test shows a good capability of the method for identifying vehicle loads from bridge response. Kim et al. [19] proposed a method to calculate vehicle bridge interaction (VBI) using extracted data from analytical and WIM systems. A two-stage identification algorithm is used for the identification of the axle force from bridge response. Table 1 presents a summary of the literature reviews on vehicle-assisted bridge SHM.

The presented review of the available literature on vehicle-assisted bridge SHM shows that most of the research to date mainly focuses on vehicle bridge interaction (VBI) -based methods and drive-by damage detection. Nonetheless, there is only one review paper for the vehicle-classification-based SHM and its focus is mainly on smart vehicle-classification methods in SHM. To the best of the authors’ knowledge, no review has been conducted to cover all vehicle-assisted techniques separately within one review paper. Some review papers have discussed vehicle-assisted bridge SHM, but these have focused only on VBI-based methods and drive-by damage detection. Plus, these reviews discussed limited aspects of each technique. In a paper by Malekjafarian et al. [20] a survey is carried out on next-generation smart sensing technology in bridge SHM. The findings of the research mainly focused on the application of the state-of-the-art methods in VBI and drive-by bridge SHM. In another research by Sony et al. [21] in 2019, application of smart sensing technology in SHM is reviewed. The study mainly focused on smart methods by utilizing efficient smartphones, cameras, drones, and robotic sensors. In other studies such as those by Zhu and Law [22], Tan et al. [23], and Kim et al. [24], VBI-based techniques as well as drive-by methods have been the focus of the study. Even though some reviews have been carried out, a comprehensive review of the vehicle assisted techniques in bridge SHM is still missing. The present study intends to fill this gap in the literature. Its primary aim is to offer a discussion on the vehicle assisted methods from the application point of view and to discuss the challenges hindering the real-life applicability of these methods.

In the following sections, vehicle-assisted bridge SHM methods are discussed. Afterward, VBI-based and drive-by methods as well as vehicle classification-based approaches are comprehensively addressed with the challenges ahead in market access. Finally, the prospect and the summary are given.

## 2. Vehicle-Bridge Interaction (VBI)-Based Methods

**Figure 1:**
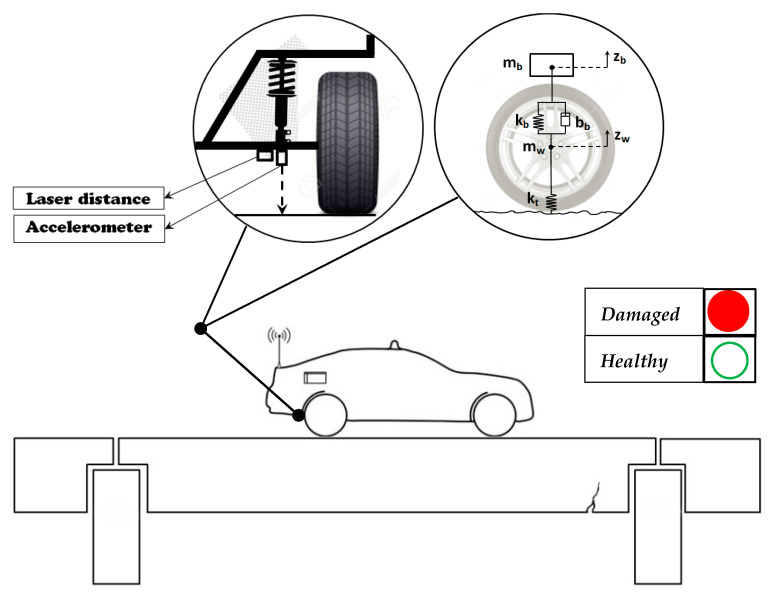
Schematic of an indirect health monitoring system for bridge structures.

Vehicle-assisted SHM methods can be divided into direct and indirect methods. In direct SHM systems, a network of sensors is deployed together to monitor a bridge structure. While in indirect monitoring systems the passing by vehicles is instrumented and the dynamic parameters of the bridge structure are obtained from the measured vehicular vibration response. These methods are also known as “drive-by” SHM methods [23]. The dynamic of moving load on bridges has been the target of many theoretical and numerical studies since the first report by Frýba [25] in the 1960s. Damage detection using conventional motion sensors such as accelerometers requires expensive sensor networks, power resources, and maintenance whereas indirect methods require one or few vibration sensors installed on the test vehicle. Hence, vehicle-assisted methods have significant advantages in the case of mobility, economy, and efficiency. Several researches are conducted to study using instrumented vehicles for damage detection [26]. Numerical simulation of a passing vehicle has been an effective tool for the analysis of VBI. Figure 1 shows the schematic of the indirect health monitoring of bridge structures. 

The vehicles in drive-by bridge health monitoring are generally equipped with accelerometers to record the acceleration time-history generated during the experiment and laser distance measuring devices to measure the vertical clearances while in motion. The quarter-car model is a simplified two-degrees-of-freedom model of the suspension system to reproduce vehicle dynamics while passing over the bridge. The quarter car model is used to demonstrate the theoretical basis of the VBI model. The vehicle is modeled as vehicle’s body *m_b_*, tire mass *m_w_* tire stiffness *k_t_*, suspension stiffness *k_b_* and, suspension damping *b_b_*. *z_w_* and *z_b_* are vertical displacements of the sprung and un-sprung masses of vehicle body and tire assemblies, respectively. r and $z_{r}$ are the road roughness and bridge deflection, respectively. When a vehicle passes over a bridge dynamic load is induced to the structure [27]. The VBI is composed of a bridge subsystem and the vehicle subsystems [28]. Though the bridge and vehicle are considered as two separate subsystems, the interaction forces at the contact points of the two subsystems make the two sets of equations coupled [29,30]. The equation of motion for sprung and unsprung masses are as Equations (1) and (2) [20].

(1) $$m_{b}{\ddot{z}}_{b}+b_{b}\left({\dot{z}}_{b}-{\dot{z}}_{w}\right)+k_{t}\left(z_{b}-z_{w}\right)=0$$

(2) $$m_{w}{\ddot{z}}_{w}-C_{s}\left({\dot{z}}_{b}-{\dot{z}}_{w}\right)-k_{b}\left(z_{b}-z_{w}\right)+k_{t}\left(z_{w}-z_{r}-r\right)=0$$

The ultimate goal of characterizing the dynamic model is to identify the modal parameters of natural frequencies, modal shapes, and damping factors in a structure. When damage occurs in a bridge structure, modal parameters change accordingly. The premise of drive-by health monitoring is that dynamic parameters of a bridge are a function of its physical properties and damage to a structure will lead to changes in dynamic parameters [31]. Existing severe damage in structure triggers an alarm to notify authorities. 

### 2.1. Stages of Damage Detection 

The identification of modal parameters plays an important role in bridge SHM. Yang et al. [32] first established the feasibility of extracting dynamic parameters of bridge structure from the response signal of a passing vehicle. A simple closed-form model of the VBI is conducted by considering a sprung mass and a simply supported beam. Since then, several researches have been published to improve the topic into its current state [20]. Tan et al. [23] proposed an algorithm to extract mode shapes and damping ratio using information from the VBI model. A numerical model of a sprung mass of a quarter-car model is adopted at a constant low speed to verify the proposed algorithm. It was demonstrated that the proposed algorithm had considerably superior performance in extracting mode shapes when compared to the algorithm presented by Yang et al. [33]. McGetrick and Kim [34] used modal parameters of the indirect approach for damage detection of an artificially damaged steel truss bridge. The result showed that the presence of damage in the structure is detectable using the proposed algorithm however it was difficult to distinguish between different damage scenarios. Tan et al. [35] and McGetrick and Kim [36] used the change in natural frequencies of a bridge using instrumented vehicles. The methods could successfully detect the presence of damage in the VBI simulation model. Chang and Kim [37] investigated the variability of bridge frequency due to parked vehicles. The frequency variability induced by parked vehicles on a VBI system was estimated by calculation. Oshima et al. [38] suggested using a heavy vehicle for excitation in addition to the scanning vehicle to yield a constant vibration on bridges. SHM methods can be broadly classified into four categories of:

1. Detection of existence;
2. Damage localization;
3. Severity assessment;
4. Prediction of the remaining life of a damaged structure.

whereas the last one is less explicitly reported in the literature [39,40]. Vibration-based damage detection (VDD) is one of the most active research areas in the field of SHM that use structural vibration to evaluate the health state of structures [20]. The underlying principle behind these techniques is that structural damage changes physical properties of a structure which in turn affects its dynamic properties [41]. VDD can be classified using the extracted features that can be any of natural frequency, damping, curvature, mode shape, and strain [20,42]. Yang and Yang [43] conducted a comprehensive review of modal identification and damage detection of bridges by indirect methods. 

#### 2.1.1. Damage Existence 

The first target in an SHM is to detect the existence of damage in a target structure. The majority of the early studies have focused specifically on using natural frequencies to distinguish damage presence in structures [44]. Lin and Yang [45] scanned the natural frequency of a sustaining bridge by an accelerometer installed in the cart towed by a truck. The natural frequencies of the cart were extracted from the recorded response using a fast Fourier transform (FFT). The field test results confirmed the applicability of indirect methods to extract the fundamental frequency of bridges. Sitton et al. [46] compared the obtained results in the literature for indirect identification manifest of natural frequencies. It was indicated that the peaks of the bridge’s natural frequency from measured vehicle response were shifted below and above the natural frequency of the bridge representing a 7% error. Cumulatively, the available literature shows that a change in natural frequency is the most effective dynamic indicator of damage in structures.

The features to identify damage presence in a structure was extended to mode shapes and damping. Obrien and Keenahan [47,48] investigated using damping of the bridge as an indicator of damage existence using drive-by bridge inspection. A two-dimensional numerical model of a three-axle truck towing a half-car trailer is used to test the effectiveness of the approach in identifying the damping of the bridge. The obtained results showed that damping can successfully detect damage in bridges with high robustness insensitive to the transverse position of the vehicle on the bridge. Keenahan et al. [49] detected the existence of damage from changes in the damping of a bridge. Theoretical model of vehicle–bridge interaction is simulated by a truck–trailer over a simply supported bridge. Subtraction of the spectra in the accelerations between the two axles is used to eliminate the effect of road profile roughness on the vehicle vibration. Though the available indirect damage identification methods using bridge damping present good potential, they face major limitations in quantification. 

Yang et al. [33] presented a theoretical algorithm to construct mode shapes of a bridge from the vibration response of test vehicles moving over the bridge. It was determined that the proposed method can offer more spatial information, with higher resolution. Important factors in constructing accurate bridge mode shapes such as road surface roughness, random traffic, and vehicle speed were studied. Oshima et al. [50] developed a method to assess the presence of different damages based on mode shapes changes. Mode shape for the bridge structure is extracted from responses of passing vehicles. Two damage scenarios were to be investigated under varying measurement noise and different road roughness. It was indicated that the damage present in structures can be recognized in severe states that incurs significant changes in modal parameters. It was stated that the developed approach has low robustness against noise. Table 2 shows some important studies on bridge SHM for damage detection in structures. 

#### 2.1.2. Damage Localization

In recent years several studies have been conducted to investigate damage localization using indirect methods. In these methods, the response signal from a drive-by vehicle is extracted and features which are sensitive to damage location are extracted. Several studies have proposed wavelet theory for damage detection and localization in indirect bridge monitoring. McGetrick et al. [34] incorporated wavelet analysis and statistical pattern recognition to both detect and locate damage in bridges. A damage feature based on wavelet coefficients is extracted and deployed on theoretical simulations, and a real bridge field experiment. The resulting damage indicators from the test vehicle and bridge showed similar patterns. Zhu and Law [55] presented a new method for damage localization using wavelet analysis. The locations of the cracks are determined from the sudden changes in the continuous wavelet transform responses. It was indicated that, using this method, locations of multiple damages can be located accurately independent of measurement noise and vehicle speed.

Signal processing and system identification methods from time and frequency domains are also used for damage localization in bridges. Lederman et al. [56] conducted a study to diagnose the location of damage by a feature extracted using principal component analysis (PCA), and kernel regression method. The response signals collected from the bridge model and vehicle passing over the model was used for analysis in a laboratory bridge model. The damage location was identified successfully using the extracted feature. Mode shapes and their derivatives appeared to have potential in damage localization in bridge structures. OBrien and Malekjafarian [57] used a damage indicator based on mode shape squares to detect the location of damage in different scenarios. Mode shapes were extracted using a short-time frequency domain decomposition method. It was stated that the method could successfully extract damage for speeds up to 8 m/s. Zhang et al. [58] proposed a new damage index based on the mode shape square. The validity of the algorithm was demonstrated by numerical simulations and simple experiments. It was indicated that more accurate results were obtained in noisy environments compared to traditional SHM algorithms. Table 3 shows some important studies conducted on localization of damages using drive-by SHM methods. 

#### 2.1.3. Severity Assessment

The derived damage feature in an indirect bridge monitoring system should be sensitive to damage existence and location but also be able to provide useful information about damage severity. Non-modal parameter-based methods such as signal processing and machine learning methods are widely used in severity assessment of damages in bridges. Wavelet transform is used by several researchers for damage severity assessment. Khorram et al. [59] attached a sensor moving load on a bridge and analyzed coefficients of continuous wavelet transform. A crack was modeled as a rotational spring from fracture mechanics. It was demonstrated that the highest magnitude of the wavelet coefficient occurs at the location of the crack. The value of the wavelet transform coefficient was correlated with the damage size. Cracks with a depth of more than 10% of the cross-section of a beam could be detected by moving sensors. Nguyen and Tran [60] presented a damage detection algorithm using wavelet transform for multi-damaged cases subjected to a moving vehicle. The dynamic response of the system was measured from a moving vehicle. Small distortions are likely to arise in the dynamic response of the system at the crack locations when the moving vehicle passes through. These small distortions can be detected by wavelet transform. The cracks’ locations are pinpointed by the peaks in wavelet transform. The obtained result for a numerical model verifies the capability of the algorithm for damages larger than 10% of the beam cross section. Table 4 shows some important studies conducted on severity assessment of damages using drive-by SHM methods.

Bridge monitoring systems that work by leveraging sensors on a passing vehicle and extracting damage features from the dynamic response of the instrumented vehicles have gained great popularity due to their lower cost. Changes in natural frequencies can be used as an indicator of damage severity of bridges. Mode shapes and their derivatives are another modal parameter used to detect and localize damage by finding discontinuities in the mode shape curvatures. However, these methods have severe limitations in severity assessment lacking experimental confirmation. Some studies that have focused on using the output-only system identification method using a combination of modal parameters including natural frequencies, mode shapes, and damping ratios have not been presented. Non-modal parameter-based methods have shown great potential in quantification of indirect SHM. However, these methods have room for performance improvement. 

### 2.2. Verification Models and Setups

A wide range of numerical simulation models, lab tests, and experimental set ups has been used for verification of the studies on the three main levels of damage detection. Various designs and settings of vehicles and bridges are presented for these indirect monitoring studies. 

#### 2.2.1. Experimental Models

It is quite interesting to see how different indirect bridge monitoring techniques work for the different bridge types and this information should be of value to researchers and engineers working with such bridges. Simply supported bridges are widely built around the world due to their advantages in terms of design and construction simplicity. In general, capabilities of indirect bridge monitoring systems for damage detection, localization, and severity assessment are studied in simply supported bridges due to its basic setting. Nakajima et al. [66] tested a trailer towed by a commercial car on a 40-m long simply-supported bridge for indirect bridge inspection. Four artificial damage scenarios were introduced into the bridge, including an intact state, an artificial crack in the girder, recovery of the crack and an artificial freezing of a hinge support. From the field moving vehicle tests, the bridge’s fundamental frequency could be successfully identified and the change in the frequency caused by the freezing of the support could be detected, verifying the feasibility and the reliability of the drive-by method using the homemade trailer. McGetrick et al. [34] incorporated wavelet analysis and statistical pattern recognition for indirect monitoring of a simply supported bridge. Discontinuity in the wavelet coefficients when the axle passes over a damaged section is considered as a damage indicator. The same pattern was obtained from recorded responses of bridge and vehicle in a steel truss bridge case-study. 

Cable-stayed bridges are an important type of bridges that are increasing in number throughout the world. However, these bridges suffer from a variety of deteriorations and loss of efficiency during their service life. Yin and Tang [67] presented a new method to detect multiple damages in a cable-stayed bridge using the dynamic response of a vehicle passing over it. Time-step integration scheme is used to solve the VBI. Simultaneous damages including deck damage and cable tension loss were introduced to a cable-stayed bridge structure and the displacement response of passing a vehicle over the intact and damaged bridge was sampled. The relative displacement response vector was decomposed using proper orthogonal decomposition. The algorithm was capable to detect multiple damage cases. Li and Zhu [68] presented field application of a drive-by parameter identification in a cable-stayed bridge. Influences of several factors faced in practical applications such as vehicle moving speed, road surface roughness, modeling uncertainties, and measurement noise are investigated. A summary of some literature on indirect monitoring of some real-world bridge structures is presented in Table 5.

#### 2.2.2. Numerical Models 

**Figure 2:**
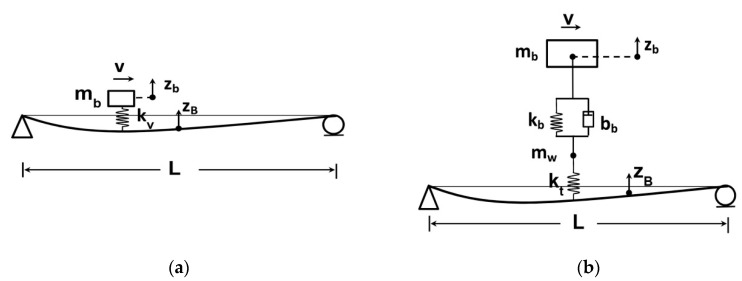
Schematic of numerical vehicles model including (**a**) moving mass and (**b**) moving sprung.

In numerical simulation, wheels of a vehicle are modeled as a point in the form of massless points [27,70], moving loads [30,71], moving masses [72,73], moving sprung masses, or other sophisticated models [74,75,76]. Figure 2 shows the configuration for moving mass and moving sprung mass models. The quarter car model is used to demonstrate the theoretical basis of the VBI model. The vehicle is modeled as vehicle’s body *m_b_*, tire mass *m_w_* tire stiffness *k_t_*, suspension stiffness *b_b_* and, suspension damping *b_b_*. *z_b_* and *z_B_* are vertical displacements of the sprung mass and bridge, respectively. The VBI in indirect bridge monitoring is shown in the form of quarter, half, or complete vehicle models. Li et al. [77] proposed using a stochastic subspace method for modal parameter identification in indirect bridge monitoring. The VBI system was simulated by a quarter-car passing over a simply-supported bridge model. Numerical results show that the proposed method was capable to estimate the bridge modal parameters. Fitzgerald et al. [78] shows the feasibility of indirect damage detection using a numerical model of a quarter-car model passing over a railway bridge. Average wavelet coefficients were proposed as a damage indicator for drive-by scour monitoring of railway bridges. It was demonstrated that the presented indicator performed quite well in normal operating conditions.

As it was shown in Equations (1) and (2), the equation of motion formulates the behavior of structure to the applied external forces in time instances. Modal parameters are a function of structural model and variation in physical and spatial properties of a structure due to deterioration or structural damage could be identified using modal analysis. Modal parameters of the bridge can be obtained by solving the equation of motions at the contact point. Acceleration response of a structure is generally used for the identification of modal properties due to its high sensitivity to change of vibration properties and richer dynamic contents [79,80]. The acceleration responses for sprung and unsprung masses are governed by the equation of motion and they can be shown in Equations (3) and (4).

(3) $${\ddot{z}}_{b}=-\frac{b_{b}\left({\dot{z}}_{b}-{\dot{z}}_{w}\right)+k_{t}\left(z_{b}-z_{w}\right)}{m_{b}}$$

(4) $${\ddot{z}}_{w}-=\frac{C_{s}\left({\dot{z}}_{b}-{\dot{z}}_{w}\right)+k_{b}\left(z_{b}-z_{w}\right)-k_{t}\left(z_{w}-z_{r}-r\right)}{m_{w}}$$

Green and Cebon [81] used a more comprehensive vehicle model capable of simulating body pitching motions using a four-degrees-of-freedom half-car model. The idea was extended by some authors through modeling full truck simulation [82,83,84]. Numerical simulation model of a real-world vehicle such as dump trucks [85] and the AASHTO HS20-44 truck [49] were used by some others. Table 6 shows the common systems used to model vehicle bridge interaction.

### 2.3. Road Surface 

Several parameters could influence the accuracy of the identified modal parameters of a bridge structure that can include bridge span length, vehicle speed, vehicle mass, damage level, and road surface roughness [36,89]. Road surface roughness is one of the most important parameters in indirect monitoring of bridges [20]. Bu et al. [90] stated that the interaction surface between vehicle and bridge has a greater influence on the variation of the modal parameters than the bridge itself. The dynamic responses of both the moving vehicle and bridge is sensitive to existing dynamic interaction forces between the moving vehicle and the structure [71]. Aside from the idealization model considered for vehicle and bridge models, the VBI is affected by the wheel–surface contact, and the adopted wheels and the track surface model [71]. 

Information on roughness or irregularity of surface profile in numerical simulation of bridges can be collected by field measurement. Using power spectral density (PSD) for generating surface roughness or track irregularity is the most common practice in numerical simulation [91]. The obtained profile by PSD tends to contain a series of hills and valleys that, in the case of the modeling wheel as a point, is unlikely to be true in reality [92]. Keenahan et al. [49] used accelerations from both axles to overcome the influence of road profile roughness on vehicle vibration. The differential spectra of the two accelerations were analyzed at the end. Chang et al. [71] proposed using a rigid disk of finite size to remedy the drawbacks of the point model. The effect of the deformation of a pneumatic tire is neglected in the mathematical model for the purpose of simplification. Tan et al. [23] investigated the effect of road surface roughness in the identification of the natural frequencies of the bridge. It was stated that the roughness has a negative impact on the identification of higher modes because of their relatively lower amplitudes. Yang and Chang [93] studied the effect of movement parameters of speed and acceleration on the quality of the extracted dynamic characteristic of a vehicle passing over a bridge.

VBI-based methods are still in the research stage and have not yet been introduced as a practical solution for real-life challenges. These methods have an analytical framework and they have been introduced for some simplified scenarios which are not expandable for analyzing large and complex models of bridge structures. 

## 3. Drive-by Damage Detection Using Mobile Sensory System

Using mobile sensors for bridge assessment through an instrumented vehicle is a promising indirect bridge inspection technique, namely “drive-by”. Drive-by technique is a promising indirect vibration-based method for bridge assessment that has emerged over the past decade. In the proposed method, instrumented vehicles were used to gather the dynamic properties of the bridge. In drive-by techniques, vehicle can be considered as both exciter and receiver [20]. 

Yang et al. [27,32] first introduced using a dynamic response of a passing vehicle to extract the dynamic properties of bridge structures. Variation in natural frequencies of a passing vehicle was used as a damage feature for the proposed drive-by technique. Since then drive-by methods have been investigated by many researchers [94,95,96]. Lin and Yang [45] used an experimental case study of passing instrumented vehicles over a highway bridge to confirm the feasibility of this method in practice. The authors employed a tractor–trailer system passing over a pre-stressed concrete bridge. It was stated that lower vehicle speeds result in a lesser influence of road surface profile and lower variation in the extracted damage feature. Using a heavy truck is found to improve frequency peak visibility. Yang et al. [97] and Chang and Kim [37] showed that the bridge frequency of a VBI system is different from the one obtained for the direct methods. In another study, Yang et al. [69] investigated the reliability of using a test cart to extract bridge frequency under various operating conditions.

Damping is another damage sensitive dynamic property of a bridge structure that is widely used in SHM [80,98]. However, the number of studies on using damping in drive-by damage detection is limited compared to frequency-based methods [20]. McGetrick et al. [99] monitored the variation of the structural damping for drive-by damage detection using various road profiles. It was shown that damage detection for smoother road profile is easier owing to the higher magnitude of peaks in the power spectral density. González et al. [100] tested the accuracy of the damping ratio in drive-by damage detection under various vehicular and structural conditions. Williams and Salawu [101] stated that the practical quantification of damping ratio is not exact and it is subject to error. Hence, several studies focused on expanding damping identification into identification of the bridge stiffness [20,100].

Mode shape of a bridge structure obtained from drive-by damage detection is another feature that can be used as a damage indicator [20,55,102]. Zhang et al. [58] proposed a method to extract an approximate estimate of structural mode shape squares from the power spectrum of a drive-by vehicle. It was mentioned that the proposed method outperforms traditional methods for damage detection in a noisy environment. Yang et al. [33] introduced a theoretical study to construct the mode shapes of a bridge from stimulus recordings of acceleration response obtained from a drive-by vehicle and natural frequency of the bridge structure. The result shows that the present approach is verified to be feasible under constant and low vehicle speeds. Oshima et al. [50] developed an indirect method to estimate mode shape from moving coordinates of the bridge structure using the singular value decomposition method. It was stated that the method needs a large number of measurement data for reliable identification of mode shapes from noisy data. Malekjafarian and OBrien [103] used short-time frequency-domain decomposition to extract the bridge mode shapes from the responses measured in a passing vehicle. Two concepts are proposed to deal with the input uncertainty caused by the road profile. Other methods are also proposed for drive-by damage detection that includes using stiffness [90,104], moving force identification [105], point impedance measured from a tapping vehicle [58], operating deflection shape curvature [106]. Table 7 shows some vehicle-assisted methods for the SHM method using vehicles as mobile sensory devices.

Overall, the indirect bridge monitoring methods using mobile sensory devices have a great potential for drive-by damage detection. Drive-by methods are still in the research and technological development phase but they are possibly viable candidates for specific applications of health monitoring of bridges. These methods suffer from the uncertainty caused by mobility parameters of vehicles and lots of influential parameters among which are the physical parameters of the vehicles and the contact surface, that significantly degrade the performances of these methods for real-life applications. The low accuracy of these methods makes them unreliable solutions as a standalone tool for the health monitoring of bridges. Moreover, due to the lack of an effective platform for the implementation of these techniques, these methods have a low capacity for commercialization and attracting business-driven investment.

## 4. Vehicle-Classification-Based Methods

The methods used in traffic engineering to derive the vehicle parameters in their moving status are defined under the term vehicle classification. Vehicle classification is a module used to categorize vehicles into several distinct classes. In these methods the vehicle could be detected by passing through a fixed sensor, passing through the monitoring area, global coverage, or a hybrid of these methods [118,119,120]. Variety of information can be extracted using the sensors and detectors which may include vehicle count, shape—height, width and length—[121], speed [122], axle weight and spacing [123], acceleration/deceleration [124], make and model [125] and number plate [126]. 

WIM is a widely used vehicle classification method for SHM of structures specifically for bridges. Deng et al. [127] used WIM for reconstructing vehicular loading in finite element (FE) model updating. The correlation between vehicular loads and damages was studied. Several sensors were installed on the bridge structure including the WIM system, global positioning systems (GPSs), strain gauges, and closed-circuit television (CCTV) cameras. Bridge WIM is an SHM method to reconstruct the loading information of a bridge structure by determining the weight of the passing over vehicles [77]. Lydon et al. [128,129] used fiber optic sensors for axle detection on an reinforced concrete bridge in Northern Ireland. The results confirmed the performance of the fiber optic sensors for gathering traffic loading information. Deng et al. [127] proposed a method to identify vehicle speed from the bridge WIM sensors. The method does not need an additional sensor for axle detection. The method was validated using numerical and experimental examples. Hou et al. [130] proposed a vision-based WIM technique to detect trucks on highway bridges and identify the loading. A clear input–output model was established for bridges to explore the correlation between the responses of different bridges to the same loading.

Suzuki et al. [131] developed a bridge WIM system to extract the acceleration response of the concrete deck slab from the velocity and weight of the passing vehicles over bridge structures. The maximum error for predicting vehicle weight was about 20% for the proposed method. Wang et al. [132] investigated vehicle classification by measuring train response of bridges obtained from WIM. The vehicle parameters such as weight, damping coefficients, and suspension stiffness can be identified using the proposed method. It is stated that the method showed acceptable robustness against noise. Dieng et al. [133] proposed a technique to determine the location of active damage zones. A combined bridge WIM technique and acoustic emission was used to monitor the health state of bridge structures under operating traffic load. 

Cantero et al. [134] introduced a virtual axle concept to detect small local damages in bridge structures. The bridge deformation is measured by bridge WIM to extract the distances between axles and axle weights. The proposed method can operate as a model-free output-only SHM system. Zhang et al. [135] presented an automated data-driven method for identification of bridge load characteristics such as the weight and speed using machine learning techniques. An experimental example by collecting WIM data from a short bridge structure was used to validate the results. Lydon et al. [136] conducted a comparative study to evaluate the performance of fiber optic and electric resistance strain sensor systems for WIM. It was observed that optical fiber networks have better performance compared to conventional methods. Ellis et al. [137] introduced a unified bridge management system to link SHM and WIM. The challenges and progresses are presented to researchers and industry.

Bridge weigh in motion (BWIM) is a type of WIM technology that is widely used for SHM of bridges. BWIM is an approach through which traffic data including speed, number of axles, axles’ spacing, and gross and axle weight of the passing vehicles are identified using a series of conventional strain gauges. BWIM is particularly suitable for short-term measurements of traffic data as it can be easily installed and detached from the bridge. The use of BWIM is preferred over the commercially available pavement WIM systems, mainly because the former offers economic benefit, requires infrequent calibration, and causes no interruption to traffic during installation. Cardini and Dewolf [138] applied BWIM through using strain gauges to gain information on the quantity and weights of the trucks crossing the highway bridge. The proposed system was able to determine the volume of trucks crossing the bridge, their gross vehicle weights, the lanes used by the trucks, and the number of overload trucks. Cantero et al. [139] proposed a BWIM-based damage identification method by introducing the concept of ‘Virtual Axle’ to derive a damage indicator. The investigations on the influence of the key parameters such as the degree and location of damage, noise levels, span lengths, and profile irregularities on the accuracy of the method show that the ‘Virtual Axle’ method can detect small local damages in statically indeterminate structures. Gonzalez and Karoumi [140] proposed a model-free damage detection method using deck accelerations response and BWIM. The proposed method is a combination of an artificial neural network and a Gaussian process that applies to railway bridges. The result of the numerical study shows that the data on the load’s position, magnitude, and speed improve the accuracy of the damage detection algorithm. Kalyankar and Uddin [141] developed a three-dimensional finite element model to estimate multi-vehicles–bridge interaction in a BWIM. Several mechanical properties of vehicles including suspension, damping, tire movement, air pressure, mass distribution on the axles, material and geometric behavior was considered in the developed 3D model for a more reliable estimation. Lydon et al. [128] developed nothing on the road (NOR) axle detection method by introducing a fiber optic BWIM system. The strain response of the live loading at various locations on the bridge was measured. The results confirmed the viability of a new strategy for axle detection. Kawakatsu et al. [142] proposed a single strain sensor-based BWIM. The obtained data were automatically optimized by consulting a surveillance camera. Satisfactory results were obtained using a single sensor application in BWIM. 

Vision-based vehicle classification is another technique that is used in health monitoring of structures. Akbar et al. [143] investigated the application of an unmanned aerial vehicle-based system to provide images of the structural site. Speeded up robust features (SURF) was used for stitching images. SURF are first reduced then transformed to align the images for final stitching. The comparison between the actual and previous view provides the structural differences. The proposed approach has also been applied on a concrete structure, and the displacement detected on the column of the structure’s backyard verified the feasibility for real-world SHM. Shan et al. [144] presented a vision-based surface flaws detection method using the Scale-invariant feature transform (SIFT) feature. In-situ tests of surface flaws are conducted on the piers of Yiqiao Bridge at Hangzhou bay. The experimental results show that the proposed method is reliable and useful for measuring surface flaws on the piers of bridge structures. Chen et al. [145]

Light detection and ranging (LiDAR) has several significant advantages over existing approaches including limited disruption to traffic, low labor requirements, and providing permanent documentations of the temporal changes of a structure. Liu et al. [146] conducted a study to explore the potential of applying LiDAR scanners for bridge-health monitoring. A surface damage detection algorithm called LiBE was presented. The LiBE algorithm differentiated information obtained from an original bridge surface through surface gradient and displacement calculation. Most of the bridge surface defects detected by the LiDAR scanner were visible to human eyes and were documented as digital photo images. Bian et al. [147] conducted a process analysis of the LiDAR bridge inspection. Several issues associated with the application of LiDAR scanning in the inspection process were pointed out. 

Other vehicle classification methods such as laser Doppler vibrometers [148], vision-based methods [149,150], Microwave radar interferometer [151] are also used for SHM of bridge structure. Table 8 presented important studies conducted on vehicle-classification-based methods for SHM.

Health monitoring of bridges using traffic information obtained using vehicle classification methods such as WIM, BWIM or strain gauges has been practiced for many years, and methods and apparatus used prior, have been modified to suit the then-current needs. However, the concept and technical principles of these methods remained largely unchanged for more than a half-century whereas the vehicles are undergoing a distinct evolution in design, and technology. The available WIM, BWM, and vision-based methods, rely on fixed-location sensors and they usually require on-site work that imposes interference with traffic. On the other hand, these methods are too expensive or subject to errors/limitations under specific situations. 

## 5. Criteria and Guidelines 

Obtaining the accurate knowledge of bridges’ behavior under real traffic load levels is one of the prerequisites for an effective condition monitoring application. However, traffic loads are entirely stochastic that makes quantitative analysis of structural load effects especially difficult [164]. Load testing offers a unique opportunity to study the real behavior of bridges [165]. 

### 5.1. Conventional Vehicle-Assisted SHM 

Load testing of bridges is as old as their construction and in the early days loading tests were carried out before the opening of the bridge [166]. The results of the loading test were an indicator that the bridge is safe enough to be opened traveling public. Sometimes it led to the collapse of the new bridge. However, the loading test is still the prerequisite before opening in some countries, such as Switzerland and Italy [166,167,168]. Nowadays the analytical method in bridge design is much more improved and more reliable methods are introduced to predict static and dynamic behavior of structures. However, the bridge loading is the most precise method to provide information about the real behavior of a bridge considering the uncertainty exposed due to the effect of the deterioration mechanism. Field testing helps engineers to have more exact values for load modeling and analysis with low uncertainty levels. On the other hand, the loading test gives more precise material properties for an existing bridge that can include resistance strength, stiffness, and impact value. Dynamic response, strain measurement, and recording displacement are of the most common data taken in a normal loading test [168]. 

Load tests are tools in getting an insight into adequacy or otherwise inadequacy of the bridge superstructure [169]. Moreover, load testing can be used for condition monitoring of constructed bridges which are faulty or which have undergone a major structural repair or strengthening [170]. The main forms of load testing of bridges are presented in Table 9. 

The desired types of measurement, location of the instrumentation, and the applied test loads should be determined by conducting a feasibility study. For instance, using global or local loading or a combination of both as the loading system should be determined. In the case of any possibility of damage to a structure, it is desirable to impose loading which is well below the threshold of tolerance by a structure [170]. Dynamic load is usually provided in a form of normal traffic, test vehicles, sudden release of deflection, sinusoidal exciter, energy input device, or the braking of a vehicle on the bridge [171]. The purpose of dynamic tests is to determine the dynamic characteristics of the bridge such as natural frequencies, mode shapes, and damping factors. Furthermore, the strain response and displacement are other parameters that are of importance that are recorded during a loading test. Bridge tests can be either for static loads that the applied load does not exceed the elastic range of structural response (or sometimes ultimate load tests) [171].

### 5.2. Detectability Range of Vibration-Based SHM

Generally, damage is defined as any change in spatial characteristics, mechanical properties, integrity, or boundary conditions of a structure which adversely affects performance, although the structure can still function satisfactorily [4,172]. Type of materials is an important factor in developing and evolving damages in structures. Concrete and steel are of the most used conventional materials in bridge construction. In concrete structures, reduction of reinforcement bar diameter, loss of bond between the steel–concrete interface, and concrete cracking are of the most reported defects [173]. On the other hand crack and corrosion are two main defects that threaten the integrity of steel structures [174,175]. 

The reduction of the cross-section in the reinforcement bar is a frequently cited damage to concrete structures. VDD methods mainly rely on loss of stiffness in structures. Since the concrete parts mainly contribute to the stiffness, deterioration of the reinforcement has little effect on natural frequency. As a result, reduction of cross-sectional area in steel reinforcement is not easy to be identified unless in structures with significant loss of reinforcement bar [176,177]. Corrosion of prestressed cables is one of the most important defects in prestressed structures. These tendons are in the form of either pre-tensioned or post-tensioned in concrete structures. Significant corrosion of prestressed cables may lead to a reduction in tensile strength and collapse of the structure. The stiffness of structures contributed predominantly by the concrete, as a result, it is difficult to detect damages of tendons using stiffness changes. Loss of prestress tendons in structures is detectable only if it is accompanied by propagation of tensile cracks [178]. Without significant loss of cross-sectional area, other damages in concrete structures such as scaling, delamination, spalling, efflorescence, pop-outs, wear and abrasion also are not detectable using conventional damage detection methods.

Steel structures are of the most frequently used structural forms in civil engineering. In steel structures, members are often connected by welding joints, bolts, or rivets. Steel structures are vulnerable to failure by fatigue and fracture. As reported, fatigue and fracture were related to 80% to 90% of the failures in steel structures [176,177]. The development of fatigue cracks can be divided into three stages of initiation, propagation, and fracture. In the initiation phase, microcracks are distributed over a structure whereas in the propagation phase the microcracks are evolved into macrocracks. In the last phase, the macrocracks grow until the structure fails. The growth of fatigue crack increases progressively in the form of the exponential function. Much fewer numbers of cycles are required to drive microcracks into fracture collapse [179]. Due to significant change in the cross-sectional area of the element, detectable variation in modal frequencies is found in the fracture phase. However, there is a high probability of masking damage by the environmental or operational noises before reaching the fracture phase. Bolt connections are frequently used joints in steel structures. Deterioration or failure of bolted joints may affect the overall integrity of a structure. In many cases, total loss or deterioration of connecting bolts is detectable using vibration-based methods. However, partial loss of bolts is not detectible due to the friction in the remaining bolts in some occasions where the connection may appear to be fixed [176,177].

As a concluding remark, one may note that the type of imperfection is a very important factor in the detectability of a damage detection system. Member loss, cracks, and bolt removal are the most probable damages that can be detected by SHM techniques and damages caused by corrosion— degradation of materials, etc.—generally cannot be distinguished by these methods. The vibration-based methods provide a powerful tool to achieve an integrated assessment of the global state of structures. However, using VDD does not necessarily mean that to expect all local and global damages in structure could be diagnosed. Perhaps these methods should be accompanied with techniques to produce a richer picture of the health state in bridge structures. A review of various testing methods and the acceptance criteria for detection damages are available in various codes and standards [180,181]. 

## 6. Future Works

The available vehicle-assisted SHM methods reviewed in this paper have valuable features and potentials that can be used by combining them with other SHM techniques such as providing complementary functions to other VDD techniques; however, using each method as a reliable standalone tool is in doubt due to several deficiencies of each method. 

VBI-based methods have an analytical framework and they have been introduced for some simplified scenarios which are not expandable for analyzing large and complex models of bridge structures. Future research in this promising area should be concentrated on developing new methods which relate to the actual complexity level of the structures and transition load in multi-vehicle cases passing through various lanes of a highway bridge. It could be facilitated by incorporating new technologies in computer engineering such as advanced software, ultrafast computing, and high capacity storage systems. Having access to real-time mobility and physical information of the vehicles passing through a bridge structure makes it possible to estimate the vehicular loading that results in the extraction of real-time behavior of the structure.

Drive-by methods are still in the research phase and could be potentially a method of choice for specific applications and to gain an overall assessment of the health state of a bridge. These methods have many limitations such as mobility parameters of vehicles which result in inconsistent results dealing with high-velocity travel speed. Moreover, there are lots of influential parameters such as physical characteristics of vehicles as well as the contact surface which have a direct impact on the obtained dynamic response. These issues significantly downgrade the applicability of these methods for real-life applications. Future work should focus on approaches to further alleviate the downsides of drive-by approaches to disentangle the complexities to make a more flexible and transparent framework.

Health monitoring of bridges using traffic information obtained using vehicle classification methods such as WIM, BWIM, or strain gauges, has been widely used for many years. The concept and technical principles of these methods remained largely unchanged for more than a half-century, whereas the vehicles are undergoing a distinct evolution in design, and technology. Nowadays autonomous and autopilot vehicles are fleeting on the roads and they are expected to revolutionize the transportation system in an unprecedented manner. Smart vehicles are equipped with various types of sensors such as cameras, LiDAR, radar, and ultrasonic sensors to observe the vehicle’s environment. Moreover, these vehicles have access to GPS, on-board unit (OBU), and a computing cloud that provides a valuable combination to be used for SHM. Hence, it is necessary to direct future efforts towards the integration of smart vehicle technology with SHM. The future of vehicle-assisted SHM is tightly linked to development and progression in other fields of industry such as automotive, electronics, and adaption of smart technologies in the transportation system. Furthermore, providing the required infrastructure and facilities is a prerequisite for a transition into intelligent health monitoring of bridges to suit the new generation of smart vehicles including autonomous or self-driving vehicles as well as autopilot ones. The adoption of smart-vehicle-assisted techniques in SHM could lead to advantages such as economic viability, ease of use and automated technology, higher reliability, decision-making capabilities, and self-sufficiency.

## 7. Conclusions

Vehicle-assisted health monitoring of bridges take advantage of vehicular data in the assessment of the health state of bridges. The obtained information could be in the form of acceleration or strain response, deformation, estimation of traffic counts for the reconstruction of the applied load, or in any other form. The methods can be grouped into three main classes of vehicle classification-based methods, drive-by and VBI-based methods. Each SHM methods have their particular assets and peculiar demerits. Depending on the objectives, available resources, and particular constraint, the optimum class of the vehicular-assisted techniques can be used for assessing the health state of bridges.

The main vehicle classification-based methods in SHM include WIM, BWIM, and vision-based techniques. BWIM and WIM have originally similar concepts and in their process, the axle parameters and gross vehicle weights can be determined during traveling over an instrumented bridge. The variety of vehicular data measured by WIM sites provides a rich source for traffic monitoring and analysis systems. WIM techniques utilize traffic-intrusive sensors and their installation and maintenance usually require on-site work that interferences with traffic. On the other hand, vehicle classification-based methods are too expensive or subject to errors/limitations under specific situations. For example, the vision-based methods may be sensitive to vehicle occlusions, weather conditions, shadows, and lighting changes. 

Drive-by techniques could be considered as a low-cost alternative for existing SHM techniques that involve direct instrumentation of the bridges with sensors and equipment for the measurement of vibration parameters of a structure. In the drive-by technique, instrumented passing vehicles over a bridge are used to gather dynamic properties of the bridge and the vehicle can be considered as both exciter and receiver. Though these methods show considerable potential, they possess some limitations such as lacking comprehensive experimental verification, and field trials. The obtained successful results have been mainly limited to bridge frequency identification under controlled conditions. The existing research addressed three main challenges for drive-by bridge monitoring that includes accuracy dependency of these methods on-road profile, speed, and environmental factors. VBI-based models are promising analytical techniques for bridge SHM under moving loads. The simplified finite element model of vehicle–bridge interaction under the action of the moving load is simulated in static conditions. Hence, the main drawback of these models is primarily due to not taking into account the dynamics of the vehicles and also the mutual interaction between bridge and vehicle. 

Overall, this paper shows that there is a long way to go before reaching a desired level of accuracy, and robustness for vehicle-assisted bridge SHM. The prospect of these methods is tightly linked to the development of practical solutions that well-match the unique features of smart vehicle technology.

## Figures and Tables

**Table 1:** Summary of the literature reviews on vehicle-assisted bridge structural health monitoring (SHM).

Title of the Paper	References	VBI-Based Methods	Drive-by Damage Detection	Vehicle-Classification-Based SHM	Modern Vehicle-Assisted Methods
A review of indirect bridge monitoring using passing vehicles	Malekjafarian et al. [20]	✓	✓	✕	✕
A literature review of next-generation smart sensing technology in structural health monitoring	Sony et al. [21]	✕	✕	✓	✕
Structural health monitoring based on vehicle-bridge interaction: Accomplishments and challenges	Zhu and Law [22]	✓	✕	✕	✕
Extraction of Bridge Modal Parameters Using Passing Vehicle Response	Tan et al. [23]	✓	✓	✕	✕
Utilizing moving vehicles as sensors for bridge condition screening-A laboratory verification	Kim et al. [24]	✕	✓	✕	✕

**Table 2:** Summary of some literature on indirect methods used to detect damage existence in bridge structural health monitoring (SHM).

Reference	Feature Extraction	Damage Index	Model	Result
Liu et al. [51]	Stacked autoencoders	Spectrogram of acceleration	Lab-scale experimental dataset and simulation	The method is applicable in real-world structures
Cantero et al. [52]	Continuous wavelet transform	Map of coefficients	Numerical model and experimental set-up	Different vehicle suspension properties have different frequency shifts
Wang et al. [53]	Particle filter	Shifting and subtracting	Numerical and field experiment	Fundamental frequency was extracted for several driving-speed cases
Elhattab et al. [54]	Feeble feature	Frequency independent underdamped pinning stochastic resonance	Simulation model and a full-scale field test.	The algorithm could only identify the first bridge frequency
Sitton et al. [46]	-	Natural frequencies	Finite-element simulations	Observed indirect bridge frequencies had two peaks below and above the fundamental bridge frequency
Lin and Yang [45]	Fast Fourier transform	Natural frequencies	Experimental	Feasibility of scanning the fundamental frequency of the bridge using the towed cart is confirmed
Oshima et al. [50]	Singular value decomposition	Mode shape	Numerical simulations of vehicles as sprung mass	Damage can be recognized in a severe state

**Table 3:** Summary of some literature on indirect methods used to localize damage in bridge SHM.

Reference	Feature Extraction	Damage Index	Model	Result
Lederman et al. [56]	PCA and kernel regression	Frequency features used in support vector machine	A laboratory-scale experiment of a vehicle pulled by a cable	Lower error was gained for the chassis sensors compared to bridge sensor
McGetrick et al. [34]	Continuous wavelet	Wavelet coefficients	A numerical model and a steel truss bridge	Damage was located accurately in a smooth road profile and low speeds
OBrien and Malekjafarian [57]	Short time-frequency domain decomposition	Mode shape squares	Numerical case study of a half-car model	Presence and location of the damage can be detected with acceptable accuracy
Zhang et al. [58]	Short time Fourier transformation	Mode shape squares	Numerical and experimental models	Acceptable damage detection when the speed is slower than 2 m/s
Zhu and Law [55]	Wavelet analysis	Coefficients of the wavelet transform	Simulation and experiment	Locations of multiple damages can be located accurately
McGetrick and Kim [36]	Morlet wavelet	Energy of the wavelet coefficients	Theoretical and experimental models	It was found that the approach can locate damages

**Table 4:** Summary of some literature on indirect methods used for severity assessment of damages in bridge SHM.

Reference	Feature Extraction	Damage Index	Model	Result
Mei et al. [61]	Cepstrum analysis	Mel-frequency cepstral coefficients (MFCCs)	Numerical analysis and lab experiments	Useful information about existence and severity is provided
Eshkevari and Pakzad [62]	Expectation maximization (STRIDEX) and second-order blind identification	Natural frequencies and mode shapes	Numerical bridge model	Successfully identification of natural frequencies and mode shapes
Liu et al. [63]	Mapping signals to a low-dimensional latent space	Variational autoencoder (VAE)	Dataset from an in-service train	This approach outperforms a baseline model
Chen et al. [64]	Multiresolution decomposition	Subband features	A lab-scale bridge	Significant improvement in results is achieved by this method
Cerda et al. [65]	Discrete Fourier transform	Natural frequencies	Laboratory setting	Promising results were achieved
Khorram et al. [59]	Continuous wavelet transform	CWT coefficient	Cracked beam	Small cracks with 10% of the beam depth was detected
Nguyen and Tran [60]	Wavelet transform	Peaks of the wavelet transform	Numerical simulation	Small cracks with 10% of the beam depth was detected
Lederman et al. [56]	Principal component analysis and kernel regression	Frequency features used in support vector machine	A laboratory-scale experiment of a vehicle pulled by a cable	Lower error was gained for the chassis sensors compared to bridge sensor

**Table 5:** Summary of some literature for indirect monitoring of some real-world bridge structures.

Reference	Bridge Type	Purpose	Location	Result
Yin and Tang [67]	Cable-stayed bridge	Identification of cable tension loss and deck damage	Computational model of Kao Ping River Bridge in Taiwan	Differences in the relative response of the vehicle due to different damage cases can be identified by the proposed method
Li and Zhu [68]	Cable-stayed bridge	Modal identification	A bridge on Great Western Highway in New South Wales, Australia	Frequencies and mode shapes can be identified
Nakajima et al. [66]	Simply-supported composite girder bridge	Indirect bridge inspection	Uji-City, Kyoto.	Bridge’s natural frequency was successfully identified
McGetrick et al. [34]	Simply supported steel truss bridge	Indirect bridge inspection	-	Similar results were obtained for both mobile and fixed sensors
Wang et al. [53]	Simply supported box girder bridge	Extraction of bridge fundamental frequency	Tsukiji bridge, Japan	Bridge’s fundamental frequency was successfully extracted
Lin and Yang [45]	Simply supported	Scanning the fundamental bridge frequencies	Da-Wu-Lun Bridge, Taiwan	The feasibility of scanning the fundamental frequency was confirmed
Yang et al. [69]	Cable-stayed bridge	Measuring the bridge frequencies	Ping-Pu Bridge, Taipei City	Application of hand-drawn cart for field measuring of the bridge frequencies

**Table 6:** The common systems used to model vehicle bridge interaction.

Reference	Feature	Numerical Model	Extracted Parameters
Liu et al. [86]	Acceleration signals collected from a passing vehicle	Quarter-car model	Locations of the damage
Tan et al. [23]	Modal parameters of mode shapes and damping ratio	Quarter-car model	Natural frequencies, mode shapes and damping ratio
Wang et al. [87]	Structural vibration responses	Quarter vehicle model	Dynamic responses
Tan et al. [35]	Dynamic characteristics of the bridge	A quarter-car model and Half-car model	Natural frequency
McGetrick and Kim [36]	Vehicle accelerations	Half-car model	Natural frequencies
Keenahan et al. [49]	Accelerations	Two identical quarter-cars	Damping
McGetrick and Kim [34]	Accelerations	Half-car model	Natural frequencies, mode shapes and damping ratio
Pakrashi et al. [88]	Maxima values of the measured responses strain	A two-axle vehicle model	Damage
Obrien and Keenahan [47]	Changes in the power spectral density of the accelerations	Two quarter cars	Damping ratio
Obrien and Keenahan [48]	Accelerations	3-axle truck	Damping

**Table 7:** Vehicle-assisted methods for SHM method using vehicles as mobile sensory device.

Reference	Method	Feature	Extracted Parameters
Matarazzo et al. [107]	Moving smartphones	Vehicle acceleration	First three modal frequencies
Mei and Gül, [108]	Smartphones in a large number of moving vehicles	Mel-frequency cepstral coefficients and Kullback–Leibler divergence	Damage
McGetrick et al. [109]	Vehicle with fitted sensors on its axles	Global navigation satellite systems (GNSS) in the smartphone	Bridge frequency
Kim et al. [24]	Moving vehicles as moving sensors	Vehicle acceleration, vehicle’s spectral distribution pattern	Bridge-frequency, vehicle’s spectral distribution pattern, roadway surface profile
Deng and Phares, B M [110]	Load rating of bridges	Strain response of ambient traffic trucks	Load rating
Martínez et al. [111]	Drive-by monitoring	Vertical displacements of a bridge	Deflection
Yang et al. [112]	Moving loads identification	The bridge deflection and strain to moving loads	Axle loads identification
Bowe et al. [113]	Train-mounted accelerometers	Accelerations resulting from the train/track/bridge dynamic interaction	Natural frequencies
Niu [114]	Moving instrumented vehicle	Vehicle dynamic behavior	The damping ratio of the bridge
Obrien and Keenahan [48]	Instrumented tractor-trailer	Acceleration	Damping
Cerda et al. [115]	Instrumented vehicle	Signals from sensors on the vehicle (indirect monitoring)	Shifts in the fundamental frequency
Kim et al. [116]	Drive-by bridge inspection	Acceleration signals from sensors on the vehicle	Changes of dominant frequencies and damping
Kim et al. [117]	Acceleration	Vertical acceleration and gyroscopic pitching measurements of the vehicle are combined with bridge accelerations to	Vehicle positioning

**Table 8:** Vehicle-classification-based methods for SHM.

Reference	Method	Feature	Details
Mei et al. [61]	Sensors mounted on a large number of passing-by vehicles	Transformed features related to bridge damage are extracted from MFCCs and PCA	Damage identification
Martínez Otero and et al. [148]	Laser Doppler vibrometers (LDVs) installed on a vehicle	Instantaneous Curvature of the velocity (RIC)	Damage identification
Hou et al. [150]	Cameras, bridge monitoring systems, and WIM	weigh parameters	Re-identification of trucks
Kawakatsu et al. [142]	BWIM	Strain data	Speed, locus, and wheel positions
Sadeghi Eshkevari and Pakzad [152]	Moving vehicle acceleration measurements	Accelerations inside rigid vehicles	Natural frequencies and mode shapes
Liu and Yu [153]	Traffic load identification	Static and time-varying components	Weight of moving traffic loads
Kawakatsu et al. [154]	Strain prediction for bridges	Camera and strain sensors	Strain responses and bridge dynamic model
Deng et al. [127]	Detecting the speed and axles of moving vehicles	Flexural strain signal	Gross vehicle weight (GVW) and axle weights (AWs), and vehicle speed and axle spacing (AS)
Zhang et al. [151]	Microwave radar interferometer	-	Bridge dynamic responses
Khuc and Catbas, [149]	Computer Vision-Based technologies	Unit influence surface (UIS)	Damage identification
Catbas et al. [155]	Computer vision-based technologies	-	Vehicle weight estimation
Lydon et al. [128]	BWIM	Fiber optic sensors	Statistics on vehicle weight, class and frequency
Wattana and Nishio [156]	Traffic volume estimation	Dynamic response data	Traffic volume
Kalyankar and Uddin [141]	BWIM	Vehicle characteristic	Obtain vehicle parameters such as velocity, axle numbers, and their distances
Fischli et al. [157]	Fiber-optic strain gauges (FBG)	-	Number of axes per vehicle and driving speed
Fischli et al. [157]	Long-gauge strain influence line	The influence line of long-gauge strain	Axle load, wheelbase and velocity on a bridge
Gonzalez and Karoumi [140]	BWIM	Load’s position, magnitude and speed	Assessing healthy or damaged state of bridge
Cantero et al. [139]	BWIM	Bridge deformation	Detect small local damages
Cantero and González [134]	WIM	Deformation of the bridge	Axle weights and distances between axles for each vehicle
Zhang et al. [158]	WIM and VBI	Traffic loads	To identify bridge load characteristics such as the weight and speed of trucks
Augustine et al. [159]	Estimation of the applied load from measured structural response	Strain data measured at optimum locations	Estimating moving loads
Seo and Hu [160]	WIM	Network of strain sensors	-
Zong et al. [161]	WIM	WIM data and the dynamic influence line	Vehicle weight and the vehicle gaps
Chen et al. [162]	WIM	WIM data	Vehicle traffic volume, vehicle traffic composition, axle load spectrum and gross vehicle weight spectrum
Xu et al. [163]	WIM	WIM data	Traffic condition and vehicle loading
Cardini and Dewolf [138]	BWIM	WIM data	Gross vehicle weights of trucks crossing steel girder bridges

**Table 9:** The available load testing methods used for SHM of bridges [170].

Type of the Loading Test	Objective	Load Level	Potential Induced Damages
Supplementary load tests	As a complement of the analytical methods	Not exceeding normal traffic loads	No permanent structural damage
Proof loading	As a proof of satisfactory design and construction	Serviceability limit state loading.	No permanent structural damage
Proving load testing	As a proof of the load-carrying capacity of the structure	Considerably higher levels of loading than other forms of testing	Risk of irreversibly damage to bridge
Dynamic load testing	To evaluate the performance of a structure	Ambient or forced vibrations	No permanent structural damage
